# Skeletal Muscle Echo Intensity Values Differ Significantly across Ultrasound Parameter Settings

**DOI:** 10.3390/life14030291

**Published:** 2024-02-22

**Authors:** Aldo Scafoglieri, Jona Van den Broeck, Paolo Bartocci, Erik Cattrysse, Harriët Jager-Wittenaar, Maria Cristina Gonzalez

**Affiliations:** 1 Experimental Anatomy Research Group, Faculty of Physical Education and Physiotherapy, Laarbeeklaan 103, 1090 Jette, Belgium; jona.van.den.broeck@vub.be (J.V.d.B.); erik.cattrysse@vub.be (E.C.); ha.jager@pl.hanze.nl (H.J.-W.); 2 Frailty in Aging Research Group, Faculty of Medicine and Pharmacy, Laarbeeklaan 103, 1090 Jette, Belgium; 3 Bachelor Program in Physiotherapy and Rehabilitation Sciences, SOMT University of Physiotherapy, Softwareweg 5, 3821 BN Amersfoort, The Netherlands; 4 Department of Neuroscience, Rehabilitation, Ophthalmology, Genetics, Maternal and Child Health, University of Genova—Campus Universitario di Savona, Via Magliotto, 3821 BN, 2, 17100 Savona, SV, Italy; 5 Research Group Healthy Ageing, Allied Health Care and Nursing, Hanze University of Applied Sciences Groningen, Zernikeplein 7, 9747 AS Groningen, The Netherlands; 6 Department of Gastroenterology and Hepatology, Dietetics, Radboud University Medical Center, Geert Grooteplein Zuid 10, 6525 GA Nijmegen, The Netherlands; 7 Postgraduate Program in Nutrition and Food, Federal Univeristy of Pelotas, Rua Gomes Carneiro, 01-Centro, Pelotas 96010-610, Brazil; cristinagbs@hotmail.com

**Keywords:** ultrasound, ImageJ, settings, echo intensity, muscle quality

## Abstract

Echo intensity determined by muscle ultrasound has been proposed as an efficient method for the assessment of muscle quality. The influence of changing ultrasound parameter settings on echo intensity values was assessed using a standardized approach. In this repeated measures cross-sectional study, sixteen repeated scans of rectus femoris, gracilis, and rectus abdominis were taken in 21 middle-aged persons with a portable Mindray M7 premium ultrasound machine equipped with a linear 5.0–10.0 MHz transducer. The settings of three parameters were fixed: gain, depth, and frequency. The settings of the following adjustable parameters were changed over their entire range: dynamic range, gray map, line density, persistence, and IClear. Repeated measures analyses were performed to evaluate the effect of changing the settings on echo intensity values. In all three muscles, dynamic range, gray map, and IClear correlated significantly (r_rm_-values ranging between −0.86 and 0.45) with echo intensity. In all three muscles, the echo intensity values differed significantly across the dynamic range (*p* < 0.013), gray map (*p* < 0.003), and IClear (*p* < 0.003). In middle-aged subjects, echo intensity values of lower limb and trunk muscles are significantly related to ultrasound parameters and significantly differ across their respective setting range. For the assessment of muscle quality through ultrasound, it is suggested to fix parameter settings within their midrange in order to minimize the effect of setting-dependent factors on EI values.

## 1. Introduction

Tackling the development of muscle diseases is central to global policies for the promotion of a healthy lifestyle [1]. Age-related micro- and macroscopic changes in muscle architecture and composition may accumulate over a lifetime as a result of decreased hormones, increases in inflammation, reduction in activity, and inadequate nutrition in the absence or presence of muscular disorders [2,3]. Muscle ultrasound is a well-established tool for screening, diagnosis, and follow-up of acute and chronic clinical conditions in neuromuscular and metabolic disorders [4]. The assessment of muscle quantity and quality has proven valuable in a variety of pathologies such as severe critical illness, muscular dystrophies and myositis, peripheral neuropathies, cancer, and type 2 diabetes mellitus [5,6,7,8,9]. Echo intensity (EI) as determined by B-mode ultrasound is an accepted marker for muscle quality [10,11]. Echo intensity has been associated with maximal strength, power, and functional performance [12,13,14]. Moreover, the EI can also detect neuromuscular disease progression [15,16]. For example, high EI values reflect fibrotic muscles that are rich in fat infiltration as observed in Duchenne muscular dystrophy [17]. As such, EI may be a valuable add-on method to the screening and diagnosis as well as the monitoring of neuromuscular diseases [18,19].

It is common practice in diagnostic ultrasound to adjust image parameter settings to achieve the best possible image quality [20]. However, since standardized procedures for quantifying muscle quality are lacking, comparing EI results between studies poses challenges [21]. As a result, normal values for EI of the quadriceps muscle vary largely between studies, partly due to differences in parameter settings [22,23,24]. Indeed, in order to facilitate the comparison of EI values between clinicians, more recently it has been suggested to control settings for US parameters such as gain and depth [25,26,27]. As relatively small increases (10 dB) in gain or depth (1 cm) significantly increase the EI value of the examined muscle, it has been recommended to fix both gain and image depth within and between individuals when evaluating EI [27].

However, the influence of other settings (beyond gain and depth) on measures of muscle quality is unknown so far. Based on a previous image optimization study, highlighting the most common parameters among US systems, it was hypothesized that dynamic range, gray map, line density, persistence, and IClear could all potentially affect the EI outcome [20]. Therefore, the aim of this study was to assess the influence of common US parameter settings on EI values using a standardized approach in a sample of middle-aged healthy persons.

## 2. Materials and Methods

The present study follows an analytical repeated measures cross-sectional design. It is analytical as it aims to examine the relationship between ultrasound parameters and EI. It is a repeated measures cross-sectional study because EI was assessed 16 times repeatedly at a specific point in time. Sample size calculation was based on the preliminary results of 11 participants in a feasibility study using the free software program G*Power© version 3.1.9.4 [28]. The effect size was calculated directly using the partial eta squared (η2p). For an effect size of 0.3, an alpha of 0.05, with a power of 80% and a correlation of 0.4 between the repeated measures, the sample size was estimated to be at least 20 participants. In the present study, 21 middle-aged volunteers (14 men and 7 women) were enrolled.

Prior to participating, all volunteers were instructed about the aims of the study and signed a written informed consent. All participants were adults aged 18 years or older, not affected by acute or chronic neuromuscular or metabolic disorders, such as muscular dystrophies, myositis, peripheral neuropathies, cancer, severe critical illness, type 2 diabetes mellitus, were in general good health, and had not exercised prior to the examination. The study was conducted according to the guidelines of the Declaration of Helsinki and approved by the ethics committee of the University Hospital Brussels (B.U.N. 1432020000335).

### 2.1. Ultrasound Parameters

Five US parameters that may influence EI were investigated: dynamic range (DR), gray map (GM), line density (LD), persistence (PERS), and IClear (IC) [20]. Dynamic range defines the echo strengths shown on the monitor. A low DR results in a more ‘black and white’ view, with a higher contrast enabling the detection of structure boundaries. On the other hand, a higher DR scan appears brighter and softer, giving more information about the echo patterns. The second parameter was the gray map, determining which ultrasonic signal is displayed in which grayscale (how bright/dark). Typically, an S-shaped curve is used instead of a linear correlation. This increases contrast at intensities that often occur in US images. The third parameter analyzed was line density, which determines the quality and information in the image. A change in LD impacts the frame rate. Higher LD results in an automatic lowering of the frame rate. Levels available on the machine were ‘L’ (low), ‘M’ (medium), ‘H’ (high), and ‘UH’ (ultra-high). The fourth parameter of interest was persistence, which defines how much of the previous image is taken over into the current frame. This makes the resulting image appear smoother and less wobbly. A high persistence is useful for long-time scans but might be more difficult to use for fast-moving structures such as the heart. Persistence increase may lead to signal missing. The fifth parameter was IC, whose function is to increase the image profile so as to distinguish the image boundaries.

### 2.2. Scanning Procedure and Image Analysis

Each volunteer was asked to lay on a physiotherapy treating table, in a supine position, hips and knees slightly flexed with a 10 cm thick knee roll placed under them, heels resting on the table. This position was maintained for the entire procedure. As it has previously been reported that anterior thigh and abdominal muscles are the first muscles affected by aging, the rectus femoris (RF), gracilis (GR), and the rectus abdominis (RA) muscles were scanned [29,30]. For the RF and GR muscles, a horizontal line was drawn at 50% between the greater trochanter of the femur and the lateral knee joint line of the right thigh [31]. For the RA muscle, a horizontal line starting from the linea alba was drawn in the right hemisphere of the body, 2 cm above the umbilicus [32]. A Mindray M7 premium US portable machine (© Shenzhen Mindray Bio-Medical Electronics CO., Ltd., Shenzhen, China) equipped with a linear 5.0–10.0 MHz transducer was used to perform the scanning sessions using a B-Mode setup. Images were acquired using an EFOV ultrasound method, which gathers together a sequence of B-Mode images taken from a continuous US scan, with high reliability [33,34]. All the scans were performed from medial to lateral.

The basic parameter settings for the scans were as follows: gain 60 dB, depth 6.5 cm, and frequency 10 MHz. For the other parameters, the default setup was DR 65, GM 2, LD M, PERS 0, and IC 0 (Table 1). This default setup was chosen based on the work of Steffel et al. [26], who assessed the impact of changing the gain on EI. At this point, several combinations of settings were applied as shown in Table 1. Each scan was performed by changing the image settings, one at a time, keeping the other parameter settings constant. The procedure was the same for all muscles examined. A total of 16 transverse EFOV scans were performed for each muscle, totaling 48 scans for each participant. All the scans were made by the same trained examiner. Our laboratory has recently determined the reproducibility of EI measurements by comparing the test-retest estimates for skeletal muscles obtained in 31 subjects [33]. The standard errors of measurement for the EI values of the rectus abdominis and rectus femoris muscles were 2.9 and 3.8, respectively. The minimally detectable changes at the 95% confidence level varied between 8.0 and 10.5 for the rectus abdominis and rectus femoris muscles, respectively. Special care was taken to put enough coupling gel between the skin and the transducer, to use minimal constant pressure on the skin, and to keep the transducer perpendicular to the surface in order to avoid image disturbances. Although the use of a probe fixator might minimize operator skill-dependent factors during the scanning procedure, the authors decided to simulate the clinical setting as much as possible.

**Figure 1:**
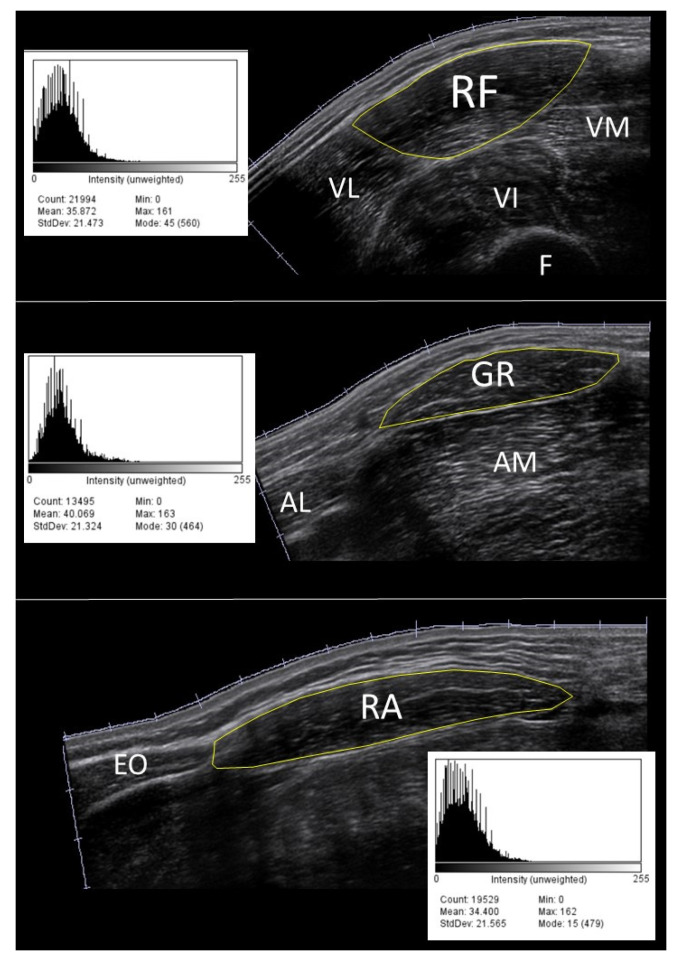
Example of the echo intensity histograms of rectus femoris (**top**), gracilis (**middle**), and rectus abdominis (**bottom**) muscle scans in a 40-year-old male volunteer using the default setup. RF = rectus femoris, VM = vastus medialis, VL = vastus lateralis, VI = vastus intermedius, F = femur, GR = gracilis, AM = adductor magnus, AL = adductor longus, RA = rectus abdominis, EO = external oblique.

All acquired images were stored on a USB drive and imported to another computer for post-processing analysis. Before data extraction, through a first visual check, a dozen images were discarded and scanned again due to the presence of artifacts, caused by generic software issues. The cross-sectional area (CSA) of the muscle was manually traced in each image using ImageJ (National Institutes of Health, Bethesda, Rockville, MD, USA). A region containing as much of the muscle as possible, avoiding intermuscular fascia and surrounding tissues, was selected [35]. The EI value of the CSA was computed based on the histogram of the image (8-bit resolution, resulting in an arbitrary unit (AU) between 0 and 256, where black = 0 and white = 255, Figure 1).

### 2.3. Statistical Analysis

Statistical analysis was performed using IBM SPSS Statistic 29.0.1 software and the stand-alone rmcorrShiny App (https://lmarusich.shinyapps.io/shiny_rmcorr/ (accessed on 15 January 2024)). The normality of data was examined with a Shapiro–Wilk test. Repeated measures correlations (rmcorr) between a given parameter and EI values were calculated. Repeated measures correlation is a statistical technique for determining the common within-individual association for paired measures assessed on two or more occasions for multiple individuals [36]. A repeated measures ANOVA was performed to evaluate the differences between EI values across the settings of a given parameter. Mauchly’s test was used to assess the assumption of sphericity. In case ANOVA was significant, a post hoc pairwise comparison with a Bonferroni adjustment was executed. Statistical significance was set at *p* < 0.05 for all tests.

## 3. Results

The characteristics of the 21 healthy participants were as follows: mean age (34.5 ± 8.7 years), height (174.9 ± 7.7 cm), and weight (72.8 ± 13.0 kg). The Shapiro–Wilk test showed normality for all EI measurements among all parameters (*p* > 0.05). The results of the repeated measures analyses are summarized in Table 2, Table 3 and Table 4.

### 3.1. Rectus Femoris Muscle

Dynamic range and EI were significantly and inversely correlated (r_rm_(83) = −0.66, 95% CI [−0.77, −0.52], *p* < 0.001). Mean EI values differed significantly across DR (F(1.7,100) = 22.5, *p* < 0.001). Post hoc pairwise comparison showed that mean EI values obtained using the default setup (at DR65) were significantly lower compared to mean EI values at DR30 (−8.0, 95% CI [−13.0, −3.0], *p* < 0.001), and higher compared to values at DR125 (4.4, 95% CI [0.7, 8.0], *p* = 0.013), and at DR150 (5.9, 95% CI [0.8, 11.0], *p* = 0.016). Gray map and EI were significantly and positively correlated (r_rm_(62) = 0.45, 95% CI [0.23, 0.63], *p* < 0.001). Mean EI values differed significantly across GM (F(3,80) = 17.5, *p* < 0.001). Post hoc pairwise comparisons also showed that the mean default setup EI values were significantly lower compared to GM4 (−9.3, 95% CI [−14.1, −4.4], *p* < 0.001) and GM8 (−10.3, 95% CI [−14.7, −5.8], *p* < 0.001) EI values, respectively. Persistence and EI were significantly and positively correlated (r_rm_(62) = 0.27, 95% CI [0.03, 0.49], *p* = 0.03). IClear and EI values were significantly and inversely correlated (r_rm_(41) = −0.79, 95% CI [−0.64, −0.88], *p* < 0.001). Mean EI values differed significantly across IC (F(1.4,60) = 34.0, *p* < 0.001). The default EI values were significantly higher compared to IC2 (4.9, 95% CI [1.9, 7.9], *p* = 0.001) and IC4 (8.7, 95% CI [5.4, 12.0], *p* < 0.001).

### 3.2. Gracilis Muscle

Dynamic range and EI were significantly and inversely correlated (r_rm_(39) = −0.43, 95% CI [−0.14, −0.65], *p* = 0.005). Mean EI values differed significantly across DR (F(4,45) = 4.9, *p* = 0.003). Gray map and EI were significantly and positively correlated (r_rm_(29) = 0.43, 95% CI [0.09, 0.68], *p* = 0.015). Mean EI values differed significantly across GM (F(3,36) = 15.4, *p* < 0.001). Post hoc pairwise comparisons also showed that the mean default setup EI values were significantly lower compared to GM8 (−10.2, 95% CI [−17.8, −2.6], *p* = 0.009) EI values. Line density and EI were significantly and positively correlated (r_rm_(29) = 0.52, 95% CI [0.20, 0.74], *p* = 0.003). Mean EI values differed significantly across LD (F(3,36) = 3.7, *p* = 0.024). IClear and EI were significantly and inversely correlated (r_rm_(19) = −0.86, 95% CI [−0.68, −0.94], *p* < 0.001). Mean EI values differed significantly across IC (F(2,27) = 31.4, *p* < 0.001). The default EI values were significantly higher compared to IC2 (7.5, 95% CI [3.1, 11.9], *p* = 0.002) and IC4 (10.8, 95% CI [6.5, 15.1], *p* < 0.001).

### 3.3. Rectus Abdominis Muscle

Dynamic range and EI values were significantly and inversely correlated (r_rm_(71) = −0.44, 95% CI [−0.61, −0.23], *p* < 0.001). Echo intensity values differed significantly across DR (F(1.5,85) = 6.2, *p* = 0.012). Gray map and EI were significantly and positively correlated (r_rm_(51) = 0.38, 95% CI [0.12, 0.59], *p* = 0.006). Echo intensity values differed significantly across GM (F(1.9,60) = 7.9, *p* = 0.002). Post hoc pairwise comparisons showed that mean EI values of the default setting were significantly lower compared to GM4 (−8.3, 95% CI [−15.4, −1.1], *p* = 0.019) and GM8 (−9.9, 95% CI [−15.4, −4.5], *p* < 0.001). IClear and EI values were significantly and inversely correlated (r_rm_(35) = −0.60, 95% CI [−0.35, −0.78], *p* < 0.001). Echo intensity values differed significantly across IC (F(1.3,51) = 9.8, *p* = 0.002). Post hoc pairwise comparisons showed that mean EI values of the default setting were significantly higher compared to IC4 (7.6, 95% CI [1.7, 13.5], *p* = 0.002).

## 4. Discussion

This study was conducted to better understand the influence of US parameter settings on EI values. According to current literature, this is the first study that looked at the effects of changing US parameter settings on EI other than gain and depth [26,27]. Our results show that changing B-mode ultrasound settings for image optimization may influence muscle EI outcomes.

Manually adjusting the DR, GM, and IC settings of an EFOV scan may change the EI value of skeletal muscle. Weak to moderate negative correlations between DR and EI values in all three muscles were found. The correlation was stronger for the rectus femoris compared to the gracilis and rectus abdominis muscles. This may be explained by the fact that the mean differences in EI values across DR were larger in the rectus femoris (14.0 AU) compared to the gracilis (5.3 AU) and rectus abdominis (7.8 AU). Given the reproducibility of EI measurements in our laboratory, differences in EI smaller than the threshold of 10 AU may simply result from measurement error rather than differences in parameter settings. This suggests that the influence of DR on EI might be muscle-dependent, as differences in EI may be more pronounced in muscles that have a higher variability in echo patterns. This is in agreement with previous results that reported that at least rectus femoris seems to be influenced by non-disease-related factors, such as US parameters [37,38]. Although, according to the present literature, there are no comparative US studies that examined the density of both muscles, previous CT-based studies showed intermuscular attenuation variations in both thigh and trunk muscles as a result of differences in fat accumulation [39,40]. For the aforementioned reasons, it is suggested to fix DR at the midrange for the assessment of muscle quality at the potential expense of image quality in order to increase the comparability of EI values between studies. Of the other US parameters, GM showed weak positive correlations with EI in all three muscles. Given this is the first report to assess the effect of changing the settings of these parameters, it is difficult to make well-established inferences on EI values. The gray map follows an S-shape pattern rather than a linear one in relation to EI values [20]. This is in accordance with our findings in all three muscles, as increasing GM positively increased the EI value, with higher GM setting values (GM 4-GM 8) being similar to each other. Since the mean difference in EI value with the default setting exceeded the MDC95 of 10, it is suggested for comparability reasons to use a GM setting corresponding to the plateau phase of the S-curve. IClear setting values were strongly and negatively correlated with EI values. Although the mean differences with the default setting exceeded the MDC95 only in the gracilis muscle, it is advisable to use a midrange setting for IC when using a muscle cross-section for the determination of EI values.

The lack of standard evaluation criteria currently represents the main limitation of US in the assessment of skeletal muscle quality [41]. Our EI values obtained using the default setting of DR65 were substantially higher than those reported by Arts et al. [21] but comparable to the ones reported by Yamada et al. [23]. Although it might be speculative to attribute differences or similarities in EI values amongst studies solely to the effect of known US parameter settings, it is noticeable that both gain and DR were higher in the Arts study compared to Yamada’s and our settings. As there are no clear criteria for muscle EI value, results obtained by different US devices and settings are not comparable. As a result, the reference values that have been published until now are only usable in the clinical setting provided that the same US device with exactly the same US parameter settings are replicated [41]. Therefore, given the challenges that remain in the assessment of muscle quality for diagnosing muscle disorders [42], it is premature to establish universal reference values for specific populations. Moreover, despite the associations that have been found between EI values and functional outcomes, no attempts have been made to determine the minimally clinically important difference in muscle quality. As a result, the changes in health conditions routinely evaluated in clinical practice corresponding to EI values remain to be determined.

### 4.1. Recommendations for Clinical and Rehabilitation Practice

In applications of muscle US, it has previously been recommended to have a high degree of standardization [43]. The standardization of US measurement does include a detailed description of the device and probe, the method of measurement, and the parameter settings used for capturing the images [26,44]. Firstly, there is a consensus position to use a panoramic vision to assess the CSA of the (maximum) muscle bulk needed to quantify the EI [45]. Secondly, is recommended to describe precisely the measuring point, specific for each muscle. This is important as it has been shown that cut-off points for individual muscle CSA depend on the point of measurement [46]. So, reference values for muscle EI only make sense if they are described for each muscle separately, taking into account their physiologic and anatomic characteristics. Finally, when measuring EI, all system parameter settings need to be kept the same. This is a prerequisite for accurate diagnosis and follow-up. Unfortunately, today there is no consensus as to which parameter settings should be fixed. In summary, the adoption of standardized operating protocols for the measurement of EI will facilitate future comparative analysis of reference data [47].

### 4.2. Strengths and Limitations

A strength of this study is the use of a whole cross-section analysis of muscles instead of a region of interest (ROI)-based method. The whole cross-section approach is preferred, as it has been shown that the size and location of an ROI affect the repeatability and reproducibility in quantitative imaging [48]. It is conceivable that by considering a larger area of muscle, the measurement error related to regional intra- and extramuscular differences in muscle composition [49,50,51], as quantified by ROI placement, is reduced since muscle contours are traced manually using the ImageJ software.

A limitation of this study is the choice of muscles and the dynamic tracking location. In this study, only rectus femoris, gracilis, and rectus abdominis muscles were scanned at specific body sites. Therefore, our results cannot be generalized to other muscles nor to other standardized locations of rectus femoris, gracilis, or rectus abdominis muscle measurement. For example, it has been shown that rectus femoris muscle EI values differ significantly according to the location of measurement along the muscle belly [50]. However, it cannot be excluded that similar findings may be encountered in other skeletal muscles frequently affected by ectopic fat accumulation, especially in pathognomonic populations.

## 5. Conclusions

In this study, EI values were significantly related to US parameters in healthy middle-aged subjects. Echo intensity values differed across the DR, GM, and IC range in lower limb and trunk muscles. It is therefore suggested to fix US parameter settings within their midrange in order to minimize the effect of setting-dependent factors on EI values. These findings reconfirm the need for standardization of ultrasound echo intensity settings when applied for diagnostic purposes of muscle quality.

## Figures and Tables

**Table 1:** Default setup of the ultrasound parameters and the modified settings used during the standardized scanning protocol.

Number of Scans	Dynamic Range	Gray Map	Line Density	Persistence	IClear
Default setup					
1	65	2	M	0	0
Modified settings					
4	30–90–125–150	2	M	0	0
3	65	4-6-8	M	0	0
3	65	2	L-H-UH	0	0
3	65	2	M	2-4-6	0
2	65	2	M	0	2-4

L = low, M = medium, H = high, UH = ultra-high.

**Table 2:** Multiple comparison results for rectus femoris muscle echo intensity values across settings in 21 healthy participants.

		Repeated Measures						
	Echo Intensity	Correlation		Within-Subjects Effects			Pairwise Comparisons	
Parameter	Mean ± SD	r_rm_ (95% CI)	*p*	df	F	*p*	Mdiff (95% CI)	*p* *
Default Setup ^$^								
DR 65 GM 2 LD M PERS 0 IC 0	55.3 ± 14.6	NA		NA			NA	
Modified settings								
DR 30	63.3 ± 19.7	−0.66 (−0.52, −0.77)	<0.001	1.7	22.5	<0.001	−8.0 (−13.0, −3.0)	<0.001
DR 90	51.1 ± 11.3						4.2 (−0.3, 8.7)	0.086
DR 125	50.9 ± 10.5						4.4 (0.7, 8.0)	0.013
DR 150	49.3 ± 8.9						5.9 (0.8, 11.0)	0.016
GM 4	64.5 ± 19.3	0.45 (0.23, 0.63)	<0.001	3	17.5	<0.001	−9.3 (−14.1, −4.4)	<0.001
GM 6	58.7 ± 19.5						−3.4 (−8.3, 1.4)	0.292
GM 8	65.6 ± 16.4						−10.3 (−14.7, −5.8)	<0.001
LD L	55.7 ± 15.6	0.05 (−0.20, 0.29)	0.701	2.4	0.3	0.787	−0.4 (−4.3, 3.4)	1.000
LD H	56.3 ± 15.5						−1.0 (−4.5, 2.4)	1.000
LD UH	55.8 ± 14.6						−0.5 (−4.8, 3.8)	1.000
PERS 2	55.5 ± 14.3	0.27 (0.03, 0.49)	0.030	2	1.4	0.261	−1.0 (−3.8, 1.8)	1.000
PERS 4	55.8 ± 15.5						−1.3 (−4.7, 2.1)	1.000
PERS 6	56.4 ± 15.6						−1.9 (−5.5, 1.6)	0.775
IC 2	50.4 ± 14.9	−0.79 (−0.64, −0.88)	<0.001	1.4	34.0	<0.001	4.9 (1.9, 7.9)	0.001
IC 4	46.6 ± 15.0						8.7 (5.4, 12.0)	<0.001

SD = standard deviation, r_rm_ = repeated measures correlation, CI = confidence interval, Mdiff = mean difference, DR = dynamic range, GM = gray map, LD = line density (L = low, M = medium, H = high, UH = ultra-high), PERS = persistence, IC = IClear, ^$^ default setting = DR 65 − GM 2 − LD M − PERS 0 − IC 0, * Bonferroni corrected, NA = not appropriate.

**Table 3:** Multiple comparison results for gracilis muscle echo intensity values across settings in 21 healthy participants.

		Repeated Measures						
	Echo Intensity	Correlation		Within-Subjects Effects			Pairwise Comparisons	
Parameter	Mean ± SD	r_rm_ (95% CI)	*p*	df	F	*p*	Mdiff (95% CI)	*p* *
Default Setup ^$^								
DR 65 GM 2 LD M PERS 0 IC 0	45.2 ± 10.2	NA		NA			NA	
Modified settings								
DR 30	48.8 ± 12.3	−0.43 (−0.14, −0.65)	0.005	4	4.9	0.003	−3.6 (−11.0, 3.9)	1.000
DR 90	41.3 ± 7.2						3.9 (−1.6, 9.4)	0.291
DR 125	43.0 ± 7.4						2.2 (−3.9, 8.3)	1.000
DR 150	43.5 ± 6.0						1.7 (−5.0, 8.4)	1.000
GM 4	50.9 ± 14.1	0.43 (0.09, 0.68)	0.015	3	15.4	<0.001	−5.7 (−14.1, 2.6)	0.276
GM 6	43.5 ± 12.3						1.7 (−5.0, 8.4)	1.000
GM 8	55.3 ± 12.0						−10.2 (−17.8, −2.6)	0.009
LD L	43.3 ± 10.6	0.52 (0.20, 0.74)	0.003	3	3.7	0.024	1.8 (−2.7, 6.4)	1.000
LD H	45.3 ± 11.1						−0.1 (−4.1, 3.9)	1.000
LD UH	47.2 ± 11.2						−2.0 (−7.0, 2.9)	1.000
PERS 2	46.2 ± 10.2	0.02 (−0.33, 0.38)	0.897	3	0.2	0.896	−1.0 (−5.6, 3.6)	1.000
PERS 4	45.2 ± 10.9						−0.01 (−7.1, 7.1)	1.000
PERS 6	45.7 ± 9.7						−0.6 (−6.4, 5.3)	1.000
IC 2	37.7 ± 10.9	−0.86 (−0.68, −0.94)	<0.001	2	31.4	<0.001	7.5 (3.1, 11.9)	0.002
IC 4	34.4 ± 9.4						10.8 (6.5, 15.1)	<0.001

SD = standard deviation, r_rm_ = repeated measures correlation, CI = confidence interval, Mdiff = mean difference, DR = dynamic range, GM = gray map, LD = line density (L = low, M = medium, H = high, UH = ultra-high), PERS = persistence, IC = IClear, ^$^ default setting = DR 65 − GM 2 − LD M − PERS 0 − IC 0, * Bonferroni corrected, NA = not appropriate.

**Table 4:** Multiple comparison results for rectus abdominis muscle echo intensity values across settings in 21 healthy participants.

		Repeated Measures						
	Echo Intensity	Correlation		Within-Subjects Effects			Pairwise Comparisons	
Parameter	Mean ± SD	r_rm_ (95% CI)	*p*	df	F	*p*	Mdiff (95% CI)	*p* *
Default Setup ^$^								
DR 65 GM 2 LD M PERS 0 IC 0	46.2 ± 16.2	NA		NA			NA	
**Modified settings**								
DR 30	51.1 ± 23.6	−0.44 (−0.23, −0.61)	<0.001	1.5	6.2	0.012	−4.9 (−12.6, 2.8)	0.557
DR 90	43.1 ± 15.5						3.1 (−0.3, 6.4)	0.086
DR 125	43.8 ± 12.4						2.4 (−1.8, 6.5)	0.815
DR 150	43.3 ± 11.8						2.9 (−1.4, 7.2)	0.448
GM 4	53.6 ± 22.7	0.38 (0.12, 0.59)	0.006	1.9	7.9	0.002	−8.3 (−15.4, −1.1)	0.019
GM 6	49.4 ± 23.7						−4.1 (−12.1, 4.0)	0.859
GM 8	55.3 ± 18.1						−9.9 (−15.4, −4.5)	<0.001
LD L	45.2 ± 17.6	0.21 (−0.05, 0.45)	0.116	2.6	0.8	0.467	1.0 (−4.9, 6.9)	1.000
LD H	46.7 ± 17.9						−0.5 (−6.5, 5.4)	1.000
LD UH	47.9 ± 19.9						−1.7 (−7.5, 4.0)	1.000
PERS 2	48.6 ± 20.5	0.08 (−0.19, 0.34)	0.571	3	1.0	0.405	−2.4 (−7.6, 2.8)	1.000
PERS 4	48.5 ± 19.7						−2.3 (−8.5, 3.9)	1.000
PERS 6	47.2 ± 18.1						−1.0 (−5.0, 3.0)	1.000
IC 2	42.8 ± 18.8	−0.60 (−0.35, −0.78)	<0.001	1.3	9.8	0.002	3.4 (−1.0, 7.7)	0.169
IC 4	38.6 ± 18.5						7.6 (1.7, 13.5)	0.009

SD = standard deviation, r_rm_ = repeated measures correlation, CI = confidence interval, Mdiff = mean difference, DR = dynamic range, GM = gray map, LD = line density (L = low, M = medium, H = high, UH = ultra-high), PERS = persistence, IC = IClear, ^$^ default setting = DR 65 − GM 2 − LD M − PERS 0 −IC 0, * Bonferroni corrected, NA = not appropriate.

## Data Availability

All raw data are available upon request to the corresponding author.
